# An innovative blended learning approach using virtual patients as preparation for skills laboratory training: perceptions of students and tutors

**DOI:** 10.1186/1472-6920-13-23

**Published:** 2013-02-12

**Authors:** Ronny Lehmann, Hans Martin Bosse, Anke Simon, Christoph Nikendei, Sören Huwendiek

**Affiliations:** 1 Clinic I – General Paediatrics, Centre for Child and Adolescent Medicine, Im Neuenheimer Feld 430, Heidelberg, 69120, Germany; 2 Department of General Paediatrics, Centre for Child and Adolescent Medicine, Moorenstr. 5, Düsseldorf, 40225, Germany; 3 Department of Internal Medicine, University Hospital Heidelberg, Im Neuenheimer Feld 400, Heidelberg, 69120, Germany; 4 Department of Assessment and Evaluation, Institute of Medical Education, Faculty of Medicine, University of Bern, Konsumstrasse 13, Bern, 3010, Switzerland

**Keywords:** Medical education, Skills laboratory, Virtual patients, Blended learning

## Abstract

**Background:**

Currently only a few reports exist on how to prepare medical students for skills laboratory training. We investigated how students and tutors perceive a blended learning approach using virtual patients (VPs) as preparation for skills training.

**Methods:**

Fifth-year medical students (N=617) were invited to voluntarily participate in a paediatric skills laboratory with four specially designed VPs as preparation. The cases focused on procedures in the laboratory using interactive questions, static and interactive images, and video clips. All students were asked to assess the VP design. After participating in the skills laboratory 310 of the 617 students were additionally asked to assess the blended learning approach through established questionnaires. Tutors’ perceptions (N=9) were assessed by semi-structured interviews.

**Results:**

From the 617 students 1,459 VP design questionnaires were returned (59.1%). Of the 310 students 213 chose to participate in the skills laboratory; 179 blended learning questionnaires were returned (84.0%). Students provided high overall acceptance ratings of the VP design and blended learning approach. By using VPs as preparation, skills laboratory time was felt to be used more effectively. Tutors perceived students as being well prepared for the skills laboratory with efficient uses of time.

**Conclusion:**

The overall acceptance of the blended learning approach was high among students and tutors. VPs proved to be a convenient cognitive preparation tool for skills training.

## Background

Teaching medical procedures to large numbers of students often involve simulations in skills laboratories, which have become increasingly popular worldwide [1,2]. Skills laboratories enable the teaching of procedures in a standardised and structured manner to improve procedural skills performance [3]. Students usually get some kind of instruction and demonstration, such as use of Peyton’s four-step approach [4], training supervised by tutors, and feedback. Skills laboratory training usually includes both for students: knowledge of what should be done and how to do it, and repetitive practice under supervision. To be effective only a few students in small groups can be closely supervised at the same time to provide individual feedback which is of key importance [1]. This makes skills laboratory training quite expensive and resource intensive. To most efficiently use the precious time for skills laboratory training, and to accord with simple-to-complex-ordering of learning tasks [5,6], it is helpful if students are well prepared before attending training. Effective preparation might allow during the skills laboratory session a focus on practicing and providing repeated feedback instead of using the time for instruction. To our knowledge, mainly paper handouts are used for skills laboratory preparation of students [7]. There are few data concerning methods to prepare for skills laboratory training and their effectiveness.

Issenberg et al. reported the following 10 aspects related to a good implementation of effective learning within simulations [1]: providing feedback, repetitively practising, integration into the overall curriculum; practising with increasing levels of difficulty; adaption to multiple learning strategies; providing for clinical variation; a controlled environment; providing individualised learning; definitions of outcomes and benchmarks; validation of the learning tools. Of these recommendations, preparation for skills laboratory training was not explicitly addressed; we assume this was because of limited available data. This is consistent with a recent systematic meta-analysis by McGaghie et al. who point out that there is still little understanding regarding how different learning modalities can be integrated to best foster learning [8]. An example of integrating different learning modalities is to supplement face-to-face sessions with e-learning to enhance the effects of learning as Motschnig-Pitrik et Holzinger have shown [9].

Virtual patients (VPs) are a one-of-a-kind e-learning resource where the learner takes the role of a healthcare professional and interactively diagnoses and treats his or her patient [10]. VP approaches usually offer the possibility to embed multimedia such as video and audio clips to illustrate patient findings or procedures. A large variability of designs and approaches has been described [11]. The current literature suggests the main benefit of VPs as primarily promoting clinical reasoning skills [12]. To date VPs have mainly been reported as stand-alone teaching units. Several authors have suggested investigating how to successfully integrate e-learning in general, and VP in particular, into a curriculum [12-15].

Surprisingly, there are only a few reports available comparing different curricular integration scenarios for VPs [16-19]. These authors show that providing VPs on its own results in low acceptance and usage, while the approach is more effective when blended with face-to-face sessions. The scarce literature regarding VPs as a preparation tool mainly focuses on the preparation of communication or physical examination skills [20-22]. The authors of these studies showed that such electronic tools are well accepted among students and that they can improve performance for clinical skills and self-confidence in dealing with real patients. Edelbring et al. reported that VPs can provide a structure in the unstructured environment of a new clinical field for students to get prepared for real patient encounters [23].

Some data are available concerning various other multimedia-based tools as preparation for practising procedural skills, such as video instruction in preparation for resuscitation training or a lumbar puncture [24,25]. The ability of these methods to improve participants’ medical knowledge and technical skills, such as on cardiopulmonary resuscitation (CPR) performance, is comparable to that of tutor-led discussions. In postgraduate training, VP-like computer-based simulators seem to fill a curricular gap because they offer continuous refreshers of important resuscitation algorithms to large numbers of healthcare professionals [26].

Combining e-learning, such as the work-up of VPs, with face-to-face sessions is referred to as ‘blended learning’ [27]. Blended learning approaches aim to achieve an optimal benefit by combining different learning modalities. Because there is evidence of VPs promoting helpful cognitive preparation for soft skills learning and the physical examination [20-22], it is surprising that no data are available on the use of VPs as preparation for procedural skills training. We sought to fill this gap by investigating how students and tutors perceive the design and curricular integration of specially designed VPs used as preparation for a skills laboratory training in an innovative blended learning approach. We assumed that being cognitively prepared by VPs in self-study would result in a more efficient use of training time in a skills laboratory.

## Methods

### Paediatric rotation and participants

Fifth-year medical students (N=617) were invited to voluntary participate in skills laboratory training during their paediatric rotation at Heidelberg Medical School. The paediatric rotation consists of a four-week-module composed of lectures, bedside teaching, problem-based learning sessions, skills laboratories, and VPs. Students had to work through different kinds of VPs differentially blended with corresponding teaching activities [28]. At the end of the module student performances were evaluated by an electronic key feature exam [29] and an Objective Structured Clinical Examination (OSCE) [30].

In accordance with national practice in Germany, ethical approval was not required for this type of educational study. However, we affirmed with participants that participation was voluntary, that they would not be able to be identified from the collected data, and that no plausible harm from participation in the study could arise.

### Virtual patients

**Figure 1:**
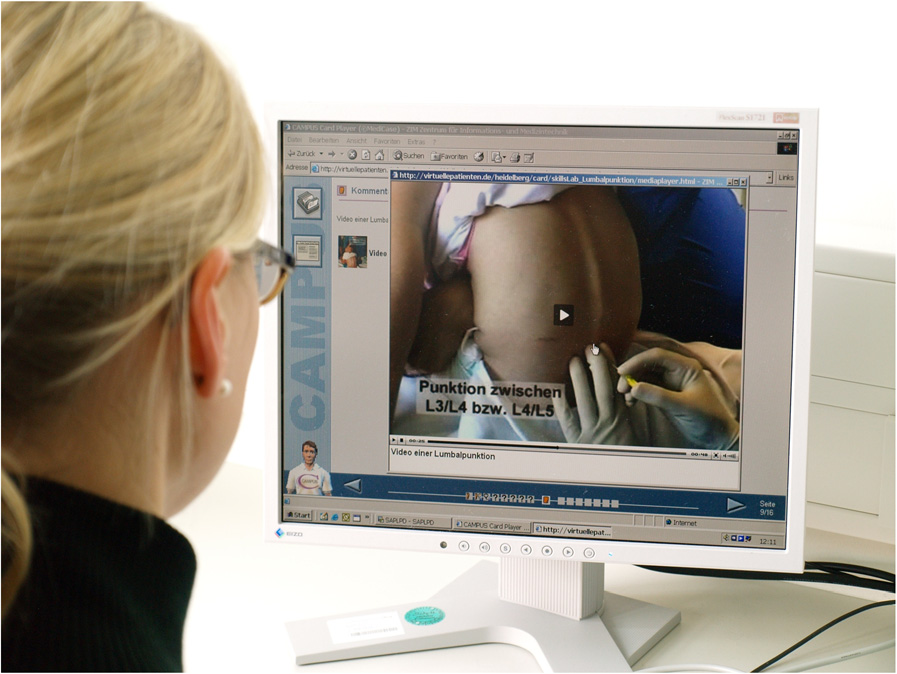
**Student using a VP for preparation of infant lumbar puncture.** The student works on a short (15 to 20 min) and interactive case of a paediatric VP. The focus is on the procedure in detail and is enhanced by media clarifications including video clips.

Four VPs were developed using the CAMPUS shell (http://www.virtual-patients.com) [31]. The VPs were designed according to published design criteria [28]. Because the cases used in the scenario had a special emphasis on preparation for skills laboratory training and less for fostering clinical reasoning skills, they were specially designed to be short (15–20 min) and the focus was on the detailed procedure enhanced by media clarifications including video clips (Figure 1). Themes and emphases of the four cases were:

– VP1: Infant with suspected meningitis, focus on lumbar puncture (informed consent, preparation, performance, and specimen care)

– VP2: Infant with suspected urinary tract infection, focus on bladder puncture (informed consent, preparation, performance, and specimen care)

– VP3: Infant with accidental asphyxia, focus on CPR (algorithm, technique)

– VP4: Toddler with cardiac arrest, focus on CPR (algorithm, technique)

The cases were accessible via our web-based learning management platform. The work-up of cases could be checked electronically for each student after completion of each VP case. For details of the cases see the characterisation of VP1 in Table 1 according to the proposed VP typology [11].

**Table 1:** **Characterization of VP1 according to **[11]

**Title:**	**Lumbar puncture**
Description:	Infant with fever comes to a paediatric emergency unit. Learner is doctor in charge and suspects meningitis. A lumbar puncture has to be indicated, prepared and performed.
Language:	German
Identifier:	SkillsLab – Lumbalpunktion
Provenance:	Lehmann, Bosse, Huwendiek, University of Heidelberg
Typical study time:	15 min
Educational level:	Undergraduate 5^th^ year
Educational modes:	Learning and formative assessment
Coverage:	Paediatrics
Objectives and outcome:	Learner will learn indications and contraindications of a lumbar puncture, what to prepare and how to perform such puncture.
Path type:	Linear string of pearls
User modality:	Single user plays the role of the paediatrician in charge
Media & resources:	Text, static graphics, video clips
Narrative use and patient focus:	Told from doctors perspective
Interactivity use:	Multiple choice questions, free text questions Overall number of cognitive interactions: 8
Feedback use:	Feedback is given to each decision immediately by comparison with the expert decision
Originating system:	CAMPUS key feature
Format:	Interactive Java-Applet inside web-browser
Integration and dependence:	Java Plug-in respectively Java Runtime Environment (JRE), CAMPUS backend, otherwise independent

### Skills laboratory training

**Figure 2:**
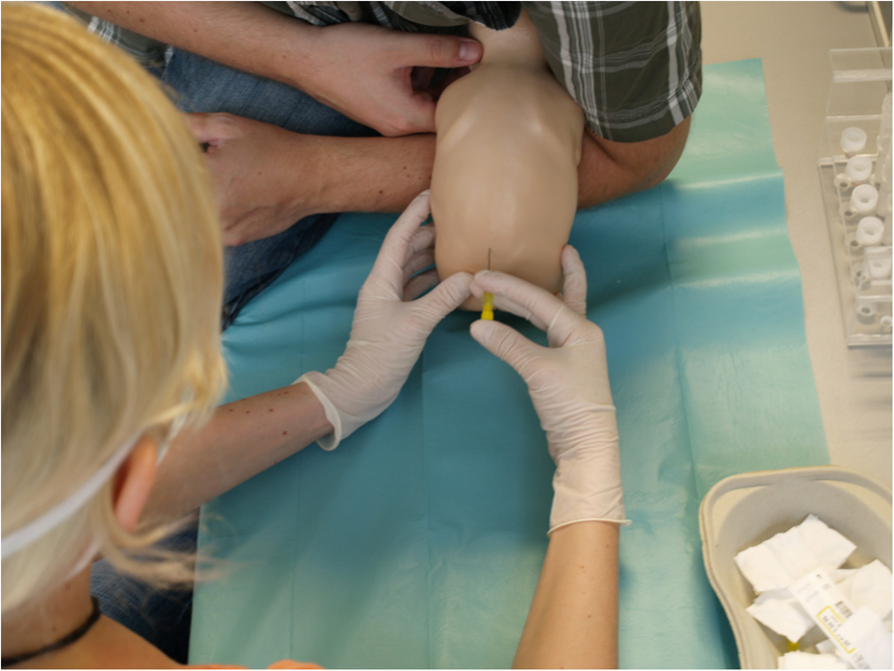
**Student practising infant lumbar puncture in the skills lab.** The student performs lumbar puncture under supervision of a tutor but without further instructions given at the beginning allowing the entire time of the training to be spent on repetitive practice under supervision with expert feedback.

The paediatric skills laboratories are embedded in a longitudinal skills laboratory curriculum of our medical school [7]. Participation in the skills laboratory training was optional to students during the study period but contents were relevant for the summative exam (OSCE) at the end of the clerkship. However, students who chose to participate were *required* to prepare themselves with the four VPs. Laboratories cover typical paediatric procedural skills, such as suprapubic bladder puncture, lumbar puncture (Figure 2), and paediatric basic life support. Supervision is provided by residents and trained senior medical student tutors. There was no instruction given at the beginning of the skills laboratory, so students were to spend the entire time of the training for repetitive practice under supervision with feedback.

### Evaluation instruments

Students’ perceptions were evaluated using two adapted instruments developed by international experts within the Electronic Virtual Patients Project (eViP, http://www.virtualpatients.eu) [32]. The first focused on the design of VPs while the second surveyed the curricular integration (blended learning) of the VPs. Because the two instruments were developed with a special focus on clinical reasoning some aspects had to be adjusted for these purposes (see below). The modified and added questions were tested for students’ understanding using the think-aloud technique and adjusted accordingly.

1. Questionnaire for students’ perceptions of VP design

The questionnaire exploring students’ perception of the VP design consisted of 15 items (12 items on a Likert scale from 1 = totally disagree to 5 = totally agree; 3 open-answer questions) clustered in the following 5 main categories: (i) authenticity (similarity to real life; 2 items), (ii) professional approach (decision making and reasoning; 3 items), (iii) coaching (embedded questions, advice, feedback, and media enhancement for the case; 4 items), (iv) learning effect (2 items), (v) overall judgement (1 item). Table 2 presents the list of items used in this study. Questions 7, 8, and 10 were modified from the original tool kit to focus on procedural skills; question 9 was added to specifically assess perceptions of the embedded media. Open-answer questions inquired about specific strengths and weaknesses of the cases, as well as about other comments.

**Table 2:** VP design questionnaire and results

**Category/items**	**Result**
*Authenticity*	
1. While working on the virtual patient I felt like making the same decisions like a physician in real life.	3.6 ± 0.7
2. While working on the virtual patient I felt like the physician in charge.	3.4 ± 0.8
*Professional approach*	
3. I was actively involved in critically challenging my image about the patient when I got new information while working on the case.	3.4 ± 0.8
4. I was actively involved in summarizing the patients’ clinical presentation in a few sentences while working on the case.	3.6 ± 0.8
5. I was actively thinking about weather my findings support or not support my differential diagnoses while working on the case.	3.5 ± 0.9
*Coaching*	
6. The level of difficulty of the case was adjusted to my level of knowledge.	3.8 ± 0.7
7. The questions I was asked while working on the case were helpful to enhance my knowledge about the procedure in this case.	4.0 ± 0.7
8. The feedback I got while working on the case were helpful to enhance my knowledge about the procedure in this case.	4.0 ± 0.7
9. The embedded media (illustrations, video clips, interactive graphics) supported my learning of the procedure.	4.1 ± 0.8
*Learning effect*	
10. After completion of the case I feel better prepared for performing this procedure on a real life patient.	3.8 ± 0.7
11. After completion of this case I feel better prepared for assuring the diagnosis and exclude important differential diagnoses on a real life patient.	3.7 ± 0.7
*Overall judgement*	
12. Overall, the case was a worthwhile learning experience.	4.0 ± 0.7
*Open ended questions*	
13. From your point of view, what are specific strengths of this case?	
14. From your point of view, what are specific weaknesses of this case?	
15. Other comments?	

All 617 students were asked to return one questionnaire for each VP (entailing a maximum of 2,468) independently of whether they chose to participate in the skills laboratory.

2. Questionnaire for students’ perceptions of the blended learning scenario

The blended learning questionnaire investigating students’ perceptions about how well VP were integrated into the curriculum consisted of 23 items (20 Likert-scaled, 3 open-answer questions; Likert scale from 1 = totally disagree to 5 = totally agree) in 5 main categories: (i) Teaching presence (the design and management of learning sequences, providing subject matter expertise, and facilitating active learning; 11 items), (ii) cognitive preparation (facilitation of focusing on practising and improvement of practical skills with efficient use of time; 3 items), (iii) social presence (ability of learners to project themselves socially and emotionally within a community of inquiry; 3 items), (iv) learning effect (2 items), (v) overall judgement (1 item). Table 3 shows the list of the items. Questions 10, 12, 13, and 18 were modified from the original tool kit to focus on the procedural skill. Questions 6 and 14 were added. Open-answer questions were about specific strengths and weaknesses of this kind of curricular integration and about suggestions for better integrations.

**Table 3:** Blended learning questionnaire and results

**Category/items**	**Result**
*Teaching presence*	
1. I felt well informed about how the virtual patients were integrated into this course.	4.1 ± 0.7
2. The chronological order of the virtual patient work and the skills laboratory was well thought out.	4.1 ± 0.7
3. The time spent on the virtual patients was well balanced with the time spent in the skills laboratory.	3.9 ± 0.8
4. The content of virtual patients and the skills laboratory complemented each other well.	4.3 ± 0.6
5. The skills laboratory gave me an insightful learning experience, which I would not have had from the virtual patients alone.	4.3 ± 0.7
6. The virtual patients gave me an insightful learning experience, which I would not have had from the skills laboratory alone.	3.6 ± 1.0
7. I think that learning with the virtual patients is important in order to do well in the final exam for this course.	3.8 ± 0.9
8. I had easy access to the virtual patients at my convenience.	3.8 ± 1.2
9. The tutors helped me to assess my learning during the skills laboratory.	3.9 ± 0.8
10. The tutors facilitated the further development of my practical skills during the skills laboratory.	4.3 ± 0.8
11. The tutors were well prepared for the skills laboratory (incl. familiarity with the virtual patients).	4.5 ± 0.7
*Cognitive preparation*	
12. I was actively involved in practical applying my newly gained insights during the skills laboratory.	4.4 ± 0.6
13. I was actively involved in refining my practical skills during the skills laboratory.	4.3 ± 0.7
14. Because of the preparation by virtual patients skills laboratory time could be used more effectively.	4.1 ± 0.8
*Social presence*	
15. During the skills laboratory the tutors created a convenient atmosphere so I could discuss about my mistakes.	4.1 ± 0.8
16. I felt a positive climate for learning during the skills laboratory.	4.4 ± 0.7
17. I felt like part of a ‘community’ during the skills laboratory.	4.3 ± 0.7
*Learning effect*	
18. The combination of virtual patients and skills laboratory enhanced my clinical practical skills.	4.0 ± 0.7
19. The combination of virtual patients and skills laboratory made me feel better prepared to care for a real life patient with this complaint.	4.1 ± 0.7
*Overall judgement*	
20. Overall, the combination of virtual patients and corresponding teaching events was a worthwhile learning experience.	4.3 ± 0.7
*Open ended questions*	
21. From your point of view, what are specific weaknesses of the overall virtual patient integration into this course?	
22. From your point of view, what are specific strengths of the overall virtual patient integration into this course?	
23. Please describe how an ideal integration of VP would look like in this context, from your point of view.	

Out of the 617 students in the study period, 310 were asked to fill in this questionnaire about the curricular integration of the blended learning scenario *after* participating in the skills laboratory.

3. Interviews of skills laboratory tutors

Skills laboratory tutors’ (N=9) perceptions were obtained by semi-structured interviews focusing on the same issues as addressed in the curricular integration questionnaire. Each interview took about 20 min. The interviews were transcribed, clustered by themes, discussed, and summarised by two of the authors (RL, SH) using content analysis [33].

### Statistical analysis

The questionnaires were analysed using SPSS version 20 (IBM Corporation). Results are given as calculated mean ± standard deviation of items per category (see Tables 2 and 3 for detailed results).

## Results

During the study period 1,459 VP design questionnaires were returned in total for all 4 VPs by 617 students (59.1%). 310 out of these 617 students were asked to evaluate the blended learning approach after participating in the skills laboratory. Of these, 213 students chose to participate in the training and 179 blended learning questionnaire forms were returned (84.0%). The results are presented below.

### VP design

**Figure 3:**
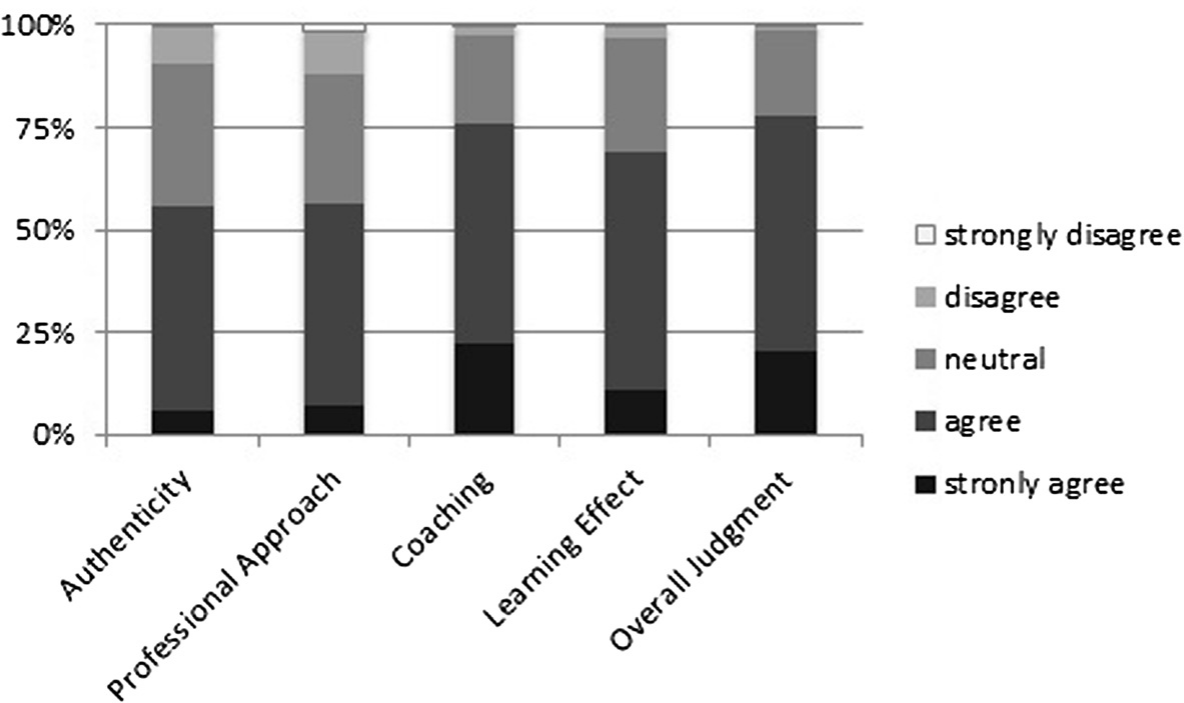
**Category results of the VP design questionnaire.** Category results of the VP design questionnaire (Likert scales from 1 – totally disagree to 5 – totally agree). Participants rated the VP design as authentic, as an acceptable professional approach, effective for coaching, and with a high learning effect. Overall judgement was very favourable.

Cases were seen as being authentic (3.5 ± 0.8) and the professional approach was considered acceptable (3.5 ± 0.8). The coaching within the cases was rated as being supportive (4.0 ± 0.7) with a good learning effect (3.8 ± 0.7). The overall judgement was 4.0 ± 0.7 (Likert scales from 1 = totally disagree to 5 = totally agree; Figure 3). Table 2 shows detailed results of all single items of the questionnaire. In the open-answer questions several students mentioned that the multimedia-based support (video clips and interactive graphics) was in particular helpful. Shortness of the case and focus on clinically relevant aspects were also mentioned as being positive, as were the presentation of typical and realistic scenarios. A few students stated that questions were too specific and that the cases were too linear with predictable results.

### Blended learning approach

**Figure 4:**
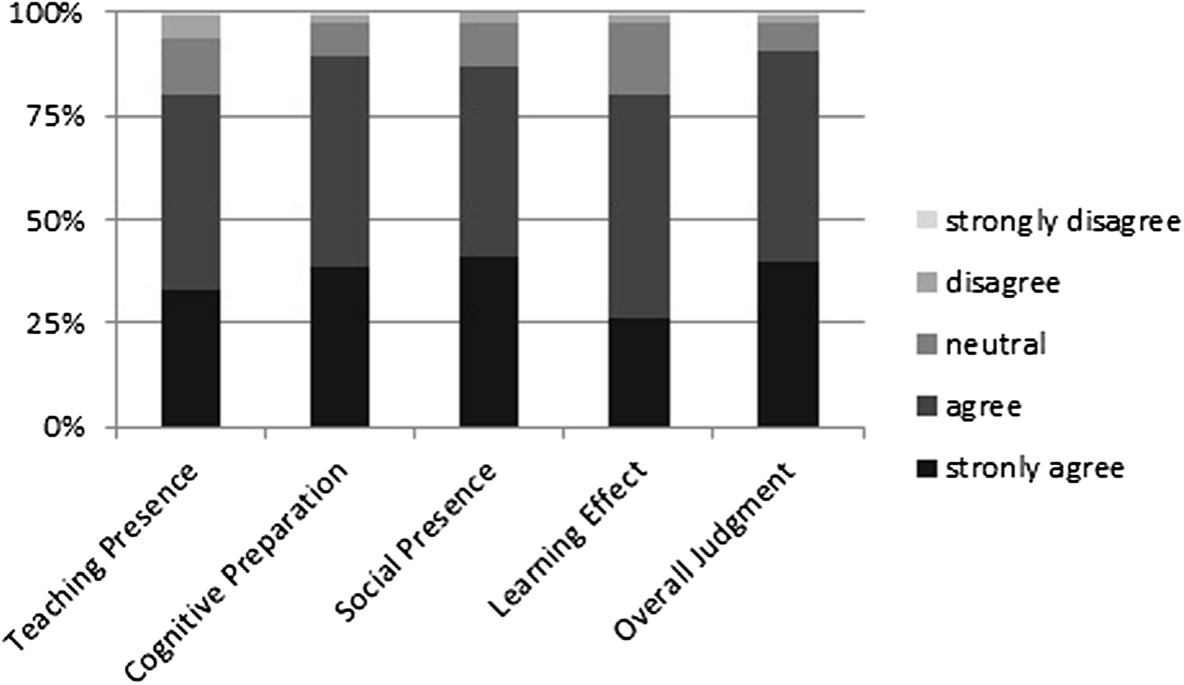
**Category results of the blended learning questionnaire.** Category results of the blended learning questionnaire (Likert scales from 1 – totally disagree to 5 – totally agree). Participants rated the blended learning scenario high for teaching presence, with a good cognitive preparation for and a good social presence in the skills lab. The learning effect was perceived effective and the overall judgement was very favourable.

The teaching presence received a very good rating by the students (4.1 ± 0.9). The cognitive preparation for (4.2 ± 0.7) and the social presence within the skills laboratory (4.3 ± 0.7) were very well perceived. Students felt a high learning effect (4.0 ± 0.7) and gave an overall judgement for the whole learning scenario of 4.3 ± 0.7 (Likert scales as before; Figure 4). Table 3 shows detailed results of all single items. The free-text entries revealed high acceptance for this kind of blended learning scenario. Several students specified that the VPs are well adjusted to the practical training and web-based training from home offers the possibility to invest time and effort individually as needed (e.g., when, where, and how long). VPs were perceived as a very good preparation.

### Tutors’ perceptions

Skills laboratory tutors perceived students as generally very well prepared for the skills laboratory training. According to the tutors, student groups usually did not need initial instructions and were able to start training right away. The tutors presumed that this was based on the interactive nature, multimedia support, showing of the complete procedure, and all given relevant information being implemented in the VP.

Tutors presumed that the interactive engagement with the VP in a very concrete way played a key role for the perceived high level of preparation. The general sequence of the procedure was usually clear to students, so only specific questions were posed. The tutors perceived the use of time as very efficient because students were hands-on the whole training time and feedback could be given during repetitive practice. The training sessions themselves were perceived as proceeding harmoniously with an open and positive atmosphere.

Tutors also perceived that the duration between the preparation with the VP and the skills training was of relevance. When the preparation was too long before the skills laboratory students’ level of preparation considerably decreased.

## Discussion

Skills laboratory training is an expensive teaching method because it takes supervision in small group tutorials with the corresponding need for numerous tutors and time slots in skills laboratories. Usually the face-to-face skills laboratory time is used for instruction, demonstrations, and repeated practice under supervision [3]. In this study we investigated how VPs as preparation for a paediatric skills laboratory were perceived by students and tutors and whether the preparation with VPs could substitute for the instruction and demonstration phase in the skills laboratory and in this way use the precious time of skills laboratory more effectively.

Overall, both students and tutors well accepted the design and the curricular integration of VPs used as preparation for skills laboratory training. Both students and tutors perceived the presented approach as a useful and effective blend. Students felt well prepared by VPs and thus could efficiently use the skills laboratory time for practical training.

Results are consistent with other reports using VPs as preparation for communication training or for specific clinical examinations. Students are generally receptive to the use of electronic learning tools [21]. For example, Holzinger et al. showed that sophisticated e-simulations can be beneficial for learning complex concepts if they are well guided and fit to students’ previous levels of knowledge [34]. The interaction with a VP prior to a real patient improved student confidence and reduced anxiety in performing breast examinations in a real patient encounter [20]. For teaching clinical skills a VP can be as effective as a standardised patient [22].

The innovative nature of our approach is to show the impact of an interactive preparation on the learning process in a skills laboratory. Other studies mainly focused on the learning outcome although the efficient use of tutor and skills laboratory time is becoming increasingly important as medical education faces restricted budgets and should thus not be neglected. In a study of communication skills training, Bosse et al. discussed costs and required resources in relation to the added value for peer role-play and standardised patients [35].

Carrero et al. reported that multimedia presentations or case-based discussions equally improved the level of cognitive skills in basic life-support training [24]. In contrast to the setting in our study the preparation with these multimedia presentations were either passive or an additional face-to-face session was required. One advantage of using VPs is to connect the attractiveness of multimedia and interactivity with the scenario of one specific patient. This makes access to the skill more transparent and understandable, and thus the clinical significance can be better understood. The presentation and explanation of a procedure within a clinical case seems to help students learn in context, to understand its clinical importance and role, to increase the perceived level of realism, and to enhance students’ involvement [36]. Similarly, Lenchus et al. reported for postgraduate training that training of procedures using a blended learning scenario which employed videos in a sequence with discussions and skills training on mannequins significantly improved participants’ knowledge and technical performance [25]. In contrast, our approach was efficient in the preparation for the skills laboratory *without* using the precious time of tutors in face-to-face discussions. In our study we assumed that we could substitute the introduction in our skills laboratory because critical points of the procedures had already been interactively worked through with questions and illustrations by videos, graphs, and figures within the VP. The participants had already seen the whole procedure in total as well as important details elaborated in the cases. This is in line with the publications of Carrero et al. and Lenchus et al. who showed that even watching a procedure is quite helpful for learning [24,25]. It is important to note that VPs do not substitute for tutors during the face-to-face training time in general, but only for the introductory information and demonstration part. When authoring VPs for such a task it is important to bear in mind that this is a special application area of VPs and design criteria might be different than in other conditions where VPs are especially used to prepare for clinical reasoning.

We think we created a good example of blended learning which combines the best of both worlds: taking advantage of interactive multimedia VPs in self-study as preparation for supervised small-group practical training sessions. This accords with cognitive load theory which suggests that reducing cognitive load during face-to-face-training results in a better learning outcome [6]. We assumed that we could reduce cognitive load during the face-to-face sessions by the preceding work-up of VPs. Strengths of this study include triangulation of judgements with using students’ feedback by means of questionnaires and tutor feedback by interviews. Because this study was a study on Kirkpatrick’s first level [37] further studies are needed to assess the effect of this kind of training and the sustainability of such an approach.

## Conclusions

Our results indicate that multimedia-enhanced VPs as described in this study offer a useful preparation for practical skills training. Through prior cognitive preparation training time was used efficiently in the skills laboratory. Future research should investigate how VPs should be designed and integrated to optimally prepare students for skills laboratory training. Furthermore, it would be interesting to compare different blended learning approaches concerning time, effort, and cost effectiveness, as well as performance improvement and sustainability.

## Competing interests

The authors declare that they have no competing interests.

## Authors’ contributions

RL and HMB developed and introduced the blended learning scenario to the curriculum. AS supported the development of the VP and accomplished the students’ evaluation. CN was responsible for the realisation within the longitudinal skills lab project at the medical school. SH was responsible for the scientific evaluation and study design. All authors read and approved the final manuscript.

## Authors’ information

Ronny Lehmann, MD, is resident and in charge for skills laboratory training at the Centre for Child and Adolescent Medicine Heidelberg, Germany. Hans Martin Bosse, MD, MME, is paediatric consultant and in charge of educational affairs at the Centre for Child and Adolescent Medicine Düsseldorf, Germany. Anke Simon, MD, is resident and jointly responsible for skills laboratory training at the Centre for Child and Adolescent Medicine Heidelberg, Germany. Christoph Nikendei, MD, MME, is internal medicine consultant and responsible for final year medical education at the Medical Hospital and the longitudinal skills laboratory curriculum at Heidelberg Medical School. Sören Huwendiek, MD, MME (Bern), is a paediatrician and was until recently chair of educational affairs at the Centre for Child and Adolescent Medicine Heidelberg, Germany, chair of the Centre for Virtual Patients and e-learning commissioner at Heidelberg Medical School. He is now head of the Department of Assessment and Evaluation at the Institute of Medical Education in Bern, Switzerland.

## Pre-publication history

The pre-publication history for this paper can be accessed here:

http://www.biomedcentral.com/1472-6920/13/23/prepub
